# Laryngeal Cancer Imaging Patterns and Agreement with Laryngoscopy Findings at Tikur Anbessa Specialized Hospital: A Retrospective Study

**DOI:** 10.4314/ejhs.v33i1.12

**Published:** 2023-01

**Authors:** Ilili Amin Aliye, Amal Saleh Nour

**Affiliations:** 1 Addis Ababa University, College of Health Sciences, Department of Radiology, Addis Ababa, Ethiopia; 2 Addis Ababa University, College of Health Sciences, Department of Radiology, Addis Ababa, Ethiopia

**Keywords:** CT, laryngeal cancer, laryngoscopy, imaging pattern

## Abstract

**Background:**

Head and neck cancer is the commonest cancer among male patients and the third commonest cancer in females at Tikur Anbessa Specialized Hospital from 1998 to 2010.

**Methods:**

A retrospective cross-sectional study of 90 patients with laryngeal mass who came to oncology and radiology departments at Tikur Anbessa specialized hospital from 2016 to 2019. Medical records were reviewed for clinical data, history, laryngoscope exam and computed tomography (CT) reports. The agreement between imaging and laryngoscope examination were analyzed.

**Results:**

The mean age of presentation was 51.5 years ±14 (SD). The primary patient complaint was hoarseness of voice 77(85.6%) followed by shortness of breath in 28(31.1%). Among 34 cases for which risk factors were indicated, 23 (67.6%) had cigarette smoking. Out of the 79 cases with laryngeal subsites described, 38 (48.1%) were transglottic, 27 (34.2%) were glottic and 12 (15.2%) were supraglottic. Extra-laryngeal spread was seen in 46(51.1%) patients and 42(46.7) were stage IVA. Out of 90 patients only 38(42.2%) patients had laryngoscope findings.

**Conclusions:**

Transglottic involvement with extra-laryngeal spread was common with advanced stage at presentation.

## Introduction

An estimated head and neck cancer incidence of up to 150,000 cases per year was made by the cancer referral center as Tikur Anbessa Specialized Hospital in Addis Ababa (1). However less attention is given for this disease which is spreading rapidly due to low awareness of the scale of the cancer burden in Ethiopia among local and international policy makers.

The pattern of cancer in Tikur Anbessa Specialized Hospital Oncology Center in Ethiopia from 1998 to 2010 showed head and neck tumors are the commonest malignancies followed by sarcomas in males and third common malignancy in females following gynecologic malignancies and breast cancer (2). A study done in Nigeria showed that the larynx is the third commonly involved site of all head and neck cancer following nasopharyngeal and sinonasal carcinoma (3). Laryngeal cancer is predominantly a disease of elderly male. In Egypt the mean age at presentation was 57.6 ± 10.5 and In India the mean age of presentation is 65 (4, 5).

There are several risk factors for laryngeal cancer as for many other cancers. These includes alcohol ingestion, cigarette smoking, and dietary factors such as red meat based diet, low vegetable and fruit diets (6). A study done in Kenya showed that Cigarette smoking and alcohol ingestion are strong risk factors for development of late stage and poorly differentiated laryngeal squamous cell carcinoma (7). The commonest clinical manifestation of laryngeal cancer is hoarseness of voice while dysphagia, neck swelling and throat pain was commoner among supra glottic cancers while hoarseness of voice was more common in glottic cancers (4).

Laryngeal subsite involvement varies amongst many studies. An Egyptian study showed that the glottis is involved only in (10.8%) of cases while (62.4%) of total patients were affected in the glottic and supraglottic regions, followed by transglottic regions in (20.7%) and supraglottic tumor occurs in 5.1% of cases[4]. In the USA, glottic carcinomas (59%) are the most common followed by supraglottic (40%) and subglottic (1%) (8).

The assessment of sub mucosal extent and invasion of adjacent structures requires cross sectional imaging. Imaging also allows the tumor to be classified according to the relevant T staging by providing important information concerning nodal metastasis, systemic metastasis and the presence of synchronous tumors (9).

The role of imaging is for staging and to guide therapy. It is also helpful in assessing post-operative complications such as radio necrosis and recurrence.

The role of cross-sectional imaging of laryngeal cancer is to define anatomical sites of involvement as supraglottic, glottic and sub glottic. Furthermore, it is used to assess tumor volume, assess relationship of the tumor to the ventricular complex, involvement of sub mucosal spaces as well as assess cervical lymph node and distant metastasis (9,10).

Imaging also evaluates extra laryngeal spread of tumors. An institutional based study of laryngectomy specimens with pre-operative contrast enhanced neck CT showed that thyroid cartilage penetration is a common route of extra laryngeal spread of glottic and supraglottic squamous cell carcinoma (11,12).

The lymph node involvement of laryngeal cancers varies according to tumor location and its extent. From all laryngeal sub sites, supraglottic cancers have higher lymph node involvement due to rich lymphatic drainage. Glottic cancers show the lowest lymphatic spread due to the lower lymphatic density in the glottic region. Subglottic cancer is rare, it is the least sub site to be involved in loco regional metastasis (13). The primary nodal drainage of the supraglottic region is to level II -IV and delphian (prelaryngeal node) for subglottic tumor (14,12).

Distant metastasis in laryngeal cancer is seen much less frequently. Amongst metastatic sites, the lung is the most common site. The mediastinum, bone and liver can also be involved (5,14).

Laryngeal cancer is staged according to AJCC which consists of T defining extent of primary tumor, N defining absence or presence of regional lymph node metastasis and M defines presence or absence of distant metastasis (15). T stage for laryngeal carcinoma is defined according to each laryngeal sub sites N and M stage is similar for all head and neck tumors except for nasopharyngeal and thyroid carcinoma with different nodal stages.

## Methods and Materials

A retrospective institutional based cross sectional study design was employed to assess the clinical and imaging pattern of laryngeal carcinoma. The study was conducted at TASH from January 2016 to July 2019. TASH is the largest referral hospital as well as the largest radiotherapy center. The source populations were all patients with laryngeal carcinoma who were referred to TASH during the study period. The study population was all patients who have cross-sectional imaging and pathology results being evaluate in the study period.

Patients whose medical records were irretrievable or incomplete were excluded from the study. The medical records of 92 patients were reviewed, 2 were excluded leaving 90 patients. The patient's demography, history, physical examination, CT and histopathology reports and laryngoscope exam were reviewed and filled on a structured questionnaire.

**Data analysis**: Data entering, coding and clearing for the quantitative data was performed using Microsoft excel and the analysis was performed with SPSS version 23. The socio-demographic and clinical characteristics of participants were computed by using simple descriptive statistics (mean, percentage, frequencies). Means and ranges were calculated from continuous variables. The agreement between imaging and laryngoscope examination was analyzed using cross-tabulation.

**Ethical consideration**: Ethical clearance was obtained from the ethics committee of the department of radiology before the commencement of the study.

## Results

We reviewed 92 medical records of which 90 fulfilled the inclusion criteria. Age ranged from 24 to 85 years with a mean age of 51.5yrs±14 (SD), 83 (92.2%) male and 7 (7.8%) were female with male to female ratio of 12:1. The primary complaint was hoarseness of voice in 77 (85.6%) patients while 28 (31.1%) had shortness of breath. Dysphagia and neck swelling were seen in 11 (12.2%) each (Table 1).

**Table 1:** Clinical features of laryngeal cancer patients at Tikur Anbessa Specialized hospital, 2017–2019

Clinical feature	Frequency	Percent
Hoarseness of voice	77	85.6
Shortness of breath	28	31.1
Dysphagia	11	12.2
Neck swelling	11	12.2
Cough	4	4.4
Upper airway obstruction	4	4.4
Chocking episode	3	3.3

Risk factor was not specified for 25 (27.8%) cases whereas for 65 (72.2%) the presence or absence of risk factors was mentioned. Out of 65 cases, 34 (52.3%) had identifiable risk factors while 31 (47.7%) had no risk factors. Among those 34 cases for which risk factors were specified, 23 (67.6%) had cigarette smoking while 12 (35.3%) had alcohol as risk factor.

The laryngeal subsite was mentioned in 79 (77.7) cases, 38 (48.1%) were transglottic, 27 (34.2%) were glottic and 12 (15.2%) were supraglottic. There was no ventricular complex involvement in 42 (46.7%) patients while 37 (41.1%) had ventricular complex involvement and for 11 (12.2%) patients, involvement of the ventricular complex was not specified.

Involvement of sub mucosal space was not specified for 53 (58.9%) cases, but in the remaining 34 (37.8%) cases which was specified, 19 (21.1%) were paraglottic, 11 (12.2%) were preglottic and the remaining 4 (4.4%) were both paraglottic and preglottic space. There was no involvement of sub mucosal space among 3 (3.3%) cases. Anterior vocal commissure was involved in 33 (36.7%) cases with no involvement in 25 (27.8%). In 32 (35.6%) cases it was not specified. Extra laryngeal spread was seen in 46 (51.1%) cases, 18 (20%) had no extra-laryngeal spread and for 26 (28.9%) cases it was not specified. Among the total 46 cases with extra-laryngeal spread, site of extension to thyroid cartilage was seen in 34 (73.9%) (Table 2).

**Table 2:** Site of extra laryngeal spread of laryngeal cancer patients at Tikur Anbessa Specialized hospital, 2017–2019

Site of extension	Frequency(%)
Thyroid Cartilage Penetration	34 (73.9)
Hypopharyngeal extension	18 (39.1)
Widening of Thyroid, Arytenoid space/Arytenoid destruction	10 (21.7)
Para vertebral, IVB	5 (10.9)
Base of tongue involvement, thyrohyoid penetration, thyroid notch penetration	3 (6.5)
Proximal Esophagus Involvement	2 (4.3)
Conus elasticus penetration	1 (2.2)

Cervical lymph nodes were described in 82 (91.1%) patients while in 8 (8.9%) it was not stated. Out of those 82 patients, 27 (32.9%) had enlarged cervical lymph nodes while 55 (67.1%) had no enlarged cervical lymph nodes. Out of the 27 cases with cervical lymph nodes involvement 10 (37%) had bilateral involvement, 12 (44.4%) had unilateral right while the remaining 5 (18.5%) had unilateral left cervical lymph node involvement.

The presence or absence of metastasis was specified for 81 (90%) of the cases while for 9 (10%) it was not specified. Out of the 81 cases, 2 (2.5%) had distant metastasis to the lung while the remaining 79 (97.5%) had no distant metastasis. Regarding the histopathology, 81 (90 %) had squamous cell carcinoma as shown on Table 3.

**Table 3:** Histopathologic pattern of laryngeal cancer patients at Tikur Anbessa Specialized hospital, 2017–2019

Histology type	Frequency (%)
SCC	81(90.0)
Carcinoma In-situ	6(6.7)
Adenocarcinoma	1(1.1)
Maltoma	1(1.1)
Undifferentiated cells	1(1.1)

Forty-two (46.7%) patients had stage IVA followed by stage III in 12 (13.3%) patients and stage II 9 (10%) as seen in Table 4. Out of 90 patients who had laryngeal carcinoma 57 (57.8%) had no laryngoscopic findings recorded on their chart. Larygoscopic finding was documented in 38 (42.2%) patients out of which 34 (89.5%) had reported specified site of involvement, 16 (47.1%) was glottic,10 (29.4%) was transglottic, 7 (20.6%) was supraglottic cancer and 1 (2.9%) patient had subglottic laryngeal cancer. There is no description on laryngoscope as to the submucosal or ventricular complex involvement. Five (5.6%) of patients with laryngeal cancer were described to have anterior commissure involvement and 8 (8.9%) had extra-laryngeal spread described as hypo pharyngeal extension.

**Table 4:** Stages of laryngeal cancer patients at Tikur Anbessa Specialized hospital, 2017–2019

Stage	Frequency	Percent
0	3	3.3
I	6	6.7
II	9	10.0
III	12	13.3
IVA	42	46.7
IVB	5	5.6
IVC	2	2.2
Not given	11	12.2
Total	90	100.0

Regarding the agreement between imaging and laryngoscope exam, for cancer located in the glottis on laryngoscopy, agreement was seen in 6 (46.2%) cases out of 13 cases. Imaging described the remaining 6 (46.2%) as being transglottic and 1 (7.7%) case as being supraglottic. Among the cases in which the location was supra-glottic on laryngoscopy, 33.3% were also supra-glottic on imaging but 66.7% were transglottic on imaging. The agreement of laryngoscopy with imaging on glottic and supraglottic locations of the cancer were 59.3% which are better than the one observed in transglottic cancers, seen on in Table 5. The overall observed agreement of laryngoscope with imaging findings as 43.3% with number of agreement expected by chance as 35.67% with Kappa value of 0.119 and SE kappa of 0.143 and 95% confidence of interval range of 0.16 to 0.399%.

**Table 5:** Comparison of the findings of imaging with that of laryngoscope in patients with laryngeal cancer at Tikur Anbessa Specialized Hospital, 2017–2019

Variable	Imaging		Laryngoscopy	
	N	%	N	%
**Laryngeal cancer**	90	100%	38	42.2%
**Site of cancer identified**	79	93.4%	34	89.5%
**Involvement of anterior commissure**	33	36.7%	5	5.6%
**Extra-laryngeal spread**	46	51%	8	8.9%

## Discussion

Among 90 patients with laryngeal carcinoma, the majority of patients were male adults with mean age 51.5 years which was slightly lower than other studies such as those done in India (65 years) and in Egypt (57.6 years) (7, 9). Our study showed that cigarette smoking and alcohol consumption were identified risk factors among laryngeal cancer patients which is similar with other studies done in Kenya and India which indicated that cigarette and alcohol as a strong risk factors among laryngeal cancer patients (9, 10). Hoarseness of voice which was the commonest clinical feature in our study is similar with clinical presentation of laryngeal cancer patients studied in Egypt. Our study also showed that shortness of breath was the second common clinical manifestation which is not in line with other studies, which can be explained due to the late presentation of patients in our study (7).

Transglottic laryngeal subsite was the most frequently involved site. The order of magnitude of laryngeal subsite involvement which was transglottic followed by glottic and supraglottic is different from studies done in USA in which glottis subsite was the commonest site followed by supraglottic cancer (6). Rather, the study matches with other studies done in developing countries where transglottic is commonly involved rather than one site (7).

Extra laryngeal spread was also assessed in this study. Thyroid cartilage penetration in 34 (73.9%) was the commonest, which is a similar finding with other studies (16).

The majority of patients with laryngeal cancer in our study did not have lymph node enlargement 55 (67.1%) while only 22(32.9%) had lymph node enlargement. These could be due to the fact that lymph node involvement is related to primary laryngeal supraglottic subsite involvement. This study had parallel findings to a study done in India where glottic cancer has less involvement of lymph node than supraglottic cancer (12). The majority of laryngeal cancer patients had no distance metastasis in this study which could be due to incomplete metastatic workup. With its limitation, this study showed that the frequently involved organ mentioned was the lung, which is similar with other studies (11), but it is different from the study done in Egypt, which indicated that liver is the only site of distant metastasis (7).

In our study, most patients had no documented laryngoscope reports on the medical record as most of the patients were referred from other hospitals to the only oncology center at TASH. Most patients who had laryngoscope findings have specified anatomic site of involvement by the laryngeal cancer but submucosal involvement as well as ventricular complex involvement was not reported on laryngoscope. Only anterior commissure and hypopharyngeal spread was reported in the minority of the cases. There was poor agreement between laryngoscopy and imaging on the anatomic location of the tumor within the larynx. Most of patients with supraglottic and glottic site involvement on laryngoscope had transglottic site involvement on imaging. Among the 38 cases of transglottic cancers on imaging, only 26.3% were identified as transglottic by laryngoscopy.

The limitation of the study was that it was a retrospective study with missing data as well as the study site being done at the only radiotherapy center in the country.

In conclusion, transglottic involvement with extra-laryngeal spread was common with advanced stage at presentation. Imaging reports were complete and provided additional detailed diagnostic information about laryngeal cancer which was not obtained with laryngoscope.

## Figures and Tables

**Figure 1:**
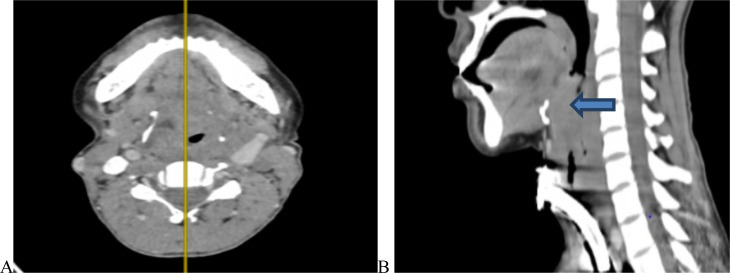
Post contrast CT findings of laryngeal cancer in 43 years old female patient, with heterogenous enhancement with oropharyngeal extension thyroid cartilage erosion(A) Transglottic pattern with hypopharyngeal extension (B)
